# Menu Audit of Vegetable-Containing Food Offering in Primary School Canteens in Sydney, Australia: A Preliminary Study

**DOI:** 10.3390/ijerph182211789

**Published:** 2021-11-10

**Authors:** Janne Beelen, Jessica E. Heffernan, Maeva Cochet-Broch, Shadia Djakovic, David Chung, Rebecca K. Golley, Astrid A. M. Poelman

**Affiliations:** 1 Sensory and Consumer Science, CSIRO Agriculture and Food, North Ryde, NSW 2113, Australia; heffernan.j89@gmail.com (J.E.H.); maeva.broch@gmail.com (M.C.-B.); astrid.poelman@csiro.au (A.A.M.P.); 2 Healthy Kids Association, 38 Oxley Street, St Leonards, NSW 2065, Australia; shadia@healthy-kids.com.au (S.D.); d.chung@healthy-kids.com.au (D.C.); 3 Caring Futures Institute, College of Nursing and Health Sciences, Flinders University, Adelaide, SA 5001, Australia; rebecca.golley@flinders.edu.au

**Keywords:** primary school, canteen, menu audit, vegetables, child nutrition, food environments, public health

## Abstract

Children’s vegetable intakes are too low, and school canteens could provide an equitable environment to improve their intake. This study aimed to develop and apply a systematic method to quantify the proportion and variety of vegetable-containing items on primary school canteen menus and examine differences between schools of different socio-economic statuses, sizes and types. Online canteen menus from 112 primary schools in Sydney, Australia, were collected, and data were extracted on a total number of items and vegetable-containing items across different menu categories. Further, data on preparation type and variety were extracted. Differences in the proportion of vegetable items between socio-economic status, school size and type were tested. On average, 80.4 ± 34.0 items were listed, with 30% of items containing vegetables. Most sandwiches (60%) and hot foods (54%) contained no vegetables. The variety of raw vegetables (4.9 ± 1.8 types) was greater than for cooked vegetables (1.3 ± 1.2 types; *p* < 0.01). Limited differences were observed by socio-economic status and school type. Small schools offered fewer vegetable-containing items than large schools. While primary school canteen menus listed a large variety of items, only one-third contained vegetables. Data from this study can be used to track changes and to develop new opportunities to increase the vegetable supply in schools.

## 1. Introduction

Vegetable consumption among children in most Western countries is below recommendations [1,2,3]. Only 6.3% of Australian children eat the recommended amount of vegetables [1], with age-dependent recommendations ranging between 2.5 and 5.5 serves per day [4], whereas children only consume two serves on average [5]. This is disconcerting as vegetables have many health benefits, including lowering the risks of heart disease, stroke and obesity [6,7,8] and eating habits track from childhood into adulthood [9].

Vegetables are predominantly consumed at dinner time [10], and consumption at different meal occasions and settings are needed in order to close the 0.5 to 3.5 serve gap between recommendations and intake. Considering the time that children spend at school, the school environment could be a viable and equitable setting to improve children’s vegetable intake. School food provision systems vary between countries [11]. For example, Swedish schools provide free meals with mandated nutritional standards, the UK has a varied and decentralised system with some schools providing free meals to students aged 4 to 7, and in Australia, meals are selected and paid for by parents if and when parents decide not to provide a home packed lunch [11]. Centralised systems ensure children are offered meals meeting nutritional requirements, while Australian children rely on canteen menus and parental choices. Therefore, opportunities to improve vegetable intake at school depend on the food provisioning system.

Over 7000 school canteens are operated across Australia by companies or parent committees [12]. These canteens function as a ‘tuck shop’, a small shop that sells meals, snacks and drinks where ordering is performed on an as-required basis, as an addition to the food being brought from home [11]. Parents, often together with their child, choose the food items their child wishes to consume using a school menu. Australian primary school children consume 39% of daily energy intake at school but only 13% of daily vegetable intake [12]. By contrast, children with a school lunch provided under the American National School Lunch Program were more likely to have vegetables than children with a lunch brought from home (29 vs. 13%) [13]. Australian children who used canteens did not consume more vegetables at school than children who brought their food from home [12]. This is surprising, as arguably canteens offer more opportunities to provide vegetables than a packed lunch.

A systematic review and meta-analysis on school food policies found most vegetable-targeted interventions led to only a very small increase in intake of 0.04 serves/day [14]. Some school food policies, such as in the USA, have specific guidelines around increasing vegetable content and diversity on menus [15], but Australian canteen policy guidelines do not. Most Australian states and territories have a traffic light system based on nutritional value [11], and New South Wales (NSW) has the Food and Drink Benchmark [16], which categorises foods as ‘Everyday’ and ‘Occasional’. Both systems have no specific focus or criteria around the presence of vegetables on menus.

Not much is known about vegetable offerings in Australian school canteens. Most research focused on adherence to policy guidelines or on the presence of discretionary foods [17,18,19,20,21,22] and did not include any quantification of the vegetable contribution on menus. An exception is Myers et al. [18], who reported that 42% of primary schools in Western Australia (WA) offered vegetable sticks, 99% offered sandwiches with vegetables and 79% offered salads. As vegetables are the most disliked food group [23], they can easily be overlooked by canteen managers in favour of other food categories to meet canteen guidelines, in particular as policy guidelines typically focus on limiting unhealthy food options. However, vegetable liking is a learned behaviour [24], and therefore normalising vegetable consumption in food environments other than at home is important both for increasing acceptance and consumption. A first step would be an inventory of the current state of play.

To our knowledge, a systematic methodology and quantification of all vegetable offerings in Australian primary school canteens are lacking. This assessment could support an increase in vegetable consumption through canteens in three ways: (1) new opportunities to increase vegetable offerings in school canteens can be identified; (2) it provides baseline audit data on current offerings, which can be tracked over time, for example, to assess the effect of new guidelines; (3) it can help determine whether segmented strategies are needed for different schools. For example, people with a lower income and socio-economic status (SES) tend to consume cheaper and energy-dense diets, often lacking vegetables and fruits [25], larger schools might have more resources to run canteens influencing food provision, and government schools need to adhere to the government’s canteen policy while non-government schools do not.

The primary aim of this study was to quantify the proportion of vegetable-containing items on online primary school canteen menus in Sydney (NSW) overall and in different menu subcategories, using a systematic methodology. The secondary aim was to compare vegetable options on menus for schools differing in SES, size and type (government versus non-government).

## 2. Materials and Methods

Canteen menus available online were downloaded from Sydney-based primary schools’ websites between July and November 2018. A stratified approach for school selection was followed. The Index of Relative Socio-Economic Advantage and Disadvantage scores (IRSEAD) were obtained from the Statistical Local Areas (SLAs) of Greater Sydney [26]. A tertile split was conducted to divide SLAs into low (IRSEAD deciles 1–5), medium (IRSEAD deciles 6–8) and high (IRSEAD 9–10) SES. Four SLAs were randomly selected per SES category using Excel’s random number generator function. Google Maps was used to identify all primary schools within each SLA, which were screened against eligibility criteria using the ‘My School’ website (www.myschool.edu.au): primary school with Kindergarten to Year 6 (K-6), or a combined primary and secondary school (K-12) if a separate primary school canteen menu was available. Schools were excluded if they were exclusively Special Learning Needs Schools or did not have a separate primary school menu.

School type (government or non-government) and enrolment size (number of students in K-6) were recorded using the ‘My School’ website. Following the Australian Bureau of Statistics classification (2015), schools were categorised as small (≤400 students), medium (401–600 students) or large (>600 students). Menus were downloaded from schools’ websites where available. Schools with only a link to an online ordering system but not a separate canteen menu were excluded from analysis. It was checked whether a menu was current by checking if it was labeled with the current school year (and season if applicable), the date it was uploaded to the website, or when school newsletters pointed at the current menu. If it could not be verified whether a menu was current, menus were not included in the analysis. The study was exempt from ethics review as no direct contact with schools was undertaken.

A systematic methodology was developed to assess the number, proportion, variety and type of vegetable-containing items on canteen menus. The World Health Organization (WHO) definition of vegetables was applied: plant parts, including tomato products, herbs and nutritionally potent vegetables in raw and cooked/processed forms, but excluding tubers and starchy vegetables [27,28]. The WHO definition does not specifically exclude legumes and pulses, but considering these are starchy, these were not counted as vegetables [28]. Variables and a coding scheme were defined based on the typical canteen menu structure and NSW Food and Drink Benchmark guidelines [16]. To verify the appropriateness and feasibility of variables and the coding scheme, a trial data extraction with 10 randomly selected menus was conducted, and input was used to finalise the structure, variable list and definitions underpinning each variable.

The following variables were manually extracted per menu:

(1) The number of total items and of vegetable-containing items on the menu overall and in subcategories. Subcategories considered were hot meals/foods, sandwiches, salads, snacks, beverages and meal deals. Flavour variants of a particular food were counted as separate items if they were listed as such. Each sandwich filling was only counted once no matter how many different types of bread (e.g., white, wholemeal) were offered, condiments and optional sauces were not counted, and each item was counted only once if listed under two different subcategories. The subcategory ‘snacks’ included items listed under ‘snacks’, ‘recess’, ‘frozen treats’ or similar. Meal deals were defined as ‘two or more items coupled for a reduced price compared to the sum of the items when purchased individually’. Many food categories listed specific ingredients used; however, many hot meals were just listed as a line item (e.g., ‘lasagne’). Where dishes did not specify the ingredients used, Taste.com.au (one of Australia’s most popular websites for recipes) was used to check the first three recipes. The item was recorded as ‘vegetable-containing’ if two recipes listed at least one vegetable as ingredient. In addition, Crunch & Sip^®^ (C&S) was included as a subcategory. C&S is a program where primary schools dedicate a specific time to eat fruit or vegetables and drink water together during class times [29,30]. Parents provide these items for their children, and some canteens offer C&S packages.

(2) For vegetable-containing items, the following data were additionally assessed: preparation type (raw or processed/cooked), variety of raw and cooked vegetables (number of different vegetables per menu) and item descriptions for hot meals/foods, snacks, meal deals and C&S.

All data were extracted manually for each individual canteen menu by a researcher and entered in Microsoft Office Excel 2013. A second researcher extracted data for 10% of the menus to check whether criteria were applied consistently.

The proportions of vegetable-containing items, overall and in subcategories, were calculated per menu. Total numbers and proportions were presented as mean ± SD, and ranges were provided. The total number of items and variety within raw and cooked vegetables was calculated per menu and tested with Paired T-tests. Differences in proportions were tested with One-way ANOVA for SES (high, medium, low) and school size (small, medium, large), and T-tests for school type (government, non-government). Post-hoc pairwise comparisons (using Bonferroni correction) were conducted when ANOVA showed a significant difference between groups. Significant differences in vegetable offering for SES and school type were also tested with ANCOVA using school size as a covariate to check whether differences could be attributable to school size. SPSS version 25 (IBM Corp., Armonk, NY, USA) was used for all statistical analyses, and a *p*-value of <0.05 was considered statistically significant.

## 3. Results

**Figure 1:**
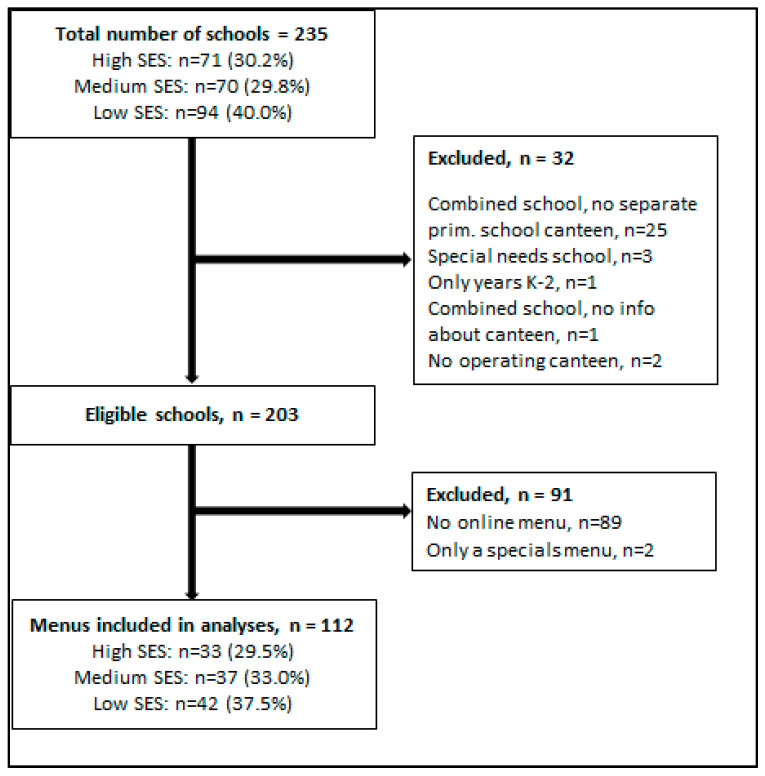
Flowchart of screening process of schools.

Using the 12 selected SLAs, 235 primary schools were identified and screened for eligibility (Figure 1), resulting in 203 eligible schools. Of these, 112 had an online menu, of which 54 (48.2%) were categorised as small, 23 (20.5%) as medium and 35 (31.3%) as large-sized schools.

**Figure 2:**
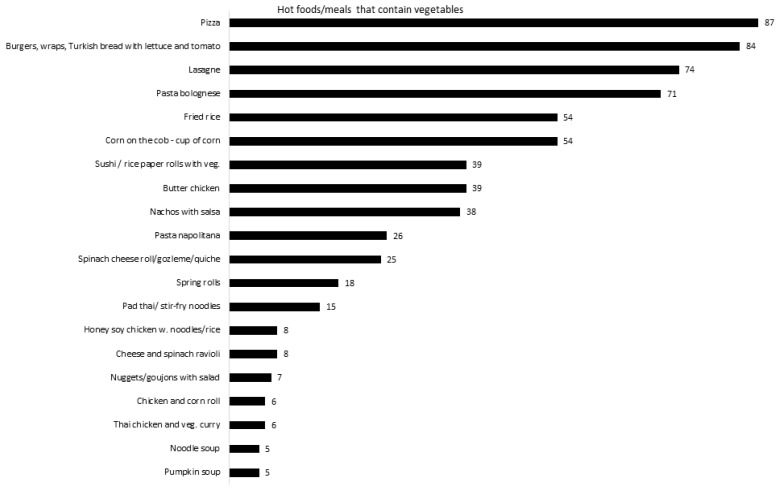
Hot foods/meals containing vegetables (the numbers represent the total number of menus that listed the item).

The total number of items listed on the 112 menus varied greatly, with the smallest menu listing 24 items and the largest menu listing 177 different items. Most menus had comparable subcategories, but small differences were found. Whereas (almost) all schools offered beverages, hot meals and sandwiches, 19 menus listed no salads, and 71 menus did not list meal deals (Table 1). Menus contained 80.4 ± 34.0 items on average overall, of which 25.7 ± 15.8 items (30.4%) contained vegetables. Almost 40% of sandwiches contained at least one vegetable by default. Forty-six percent of hot foods contained some form of vegetable. These hot foods typically included pizza, burgers, lasagne and pasta Bolognese (Figure 2). No beverages contained vegetables. Of the 23.7 ± 12.3 snacks listed, 7.9% contained vegetables. Forty-one menus listed meal deals with an average of 6.5 ± 4.9 per menu. Meal deals included two or three items, which are usually a main dish with a drink, and sometimes fruit or popcorn as a third item; 50% (3.2 ± 2.5) contained a vegetable. Only 24 menus offered C&S, of which 8 included a vegetable option. Of the salads, 22% consisted of only vegetables, 26% had optional protein components, and 52% contained protein by default. Vegetable-containing snacks were mostly raw vegetable sticks (carrot, cucumber and/or celery) with or without dips (offered on 53% of menus) and corn on the cob (on 12.5% of menus as a snack). Vegetable items offered for C&S were carrot, cucumber, celery and cherry tomatoes. Further, 9.5% of meal deals listed vegetable side dishes such as a garden salad or vegetable snack.

**Figure 3:**
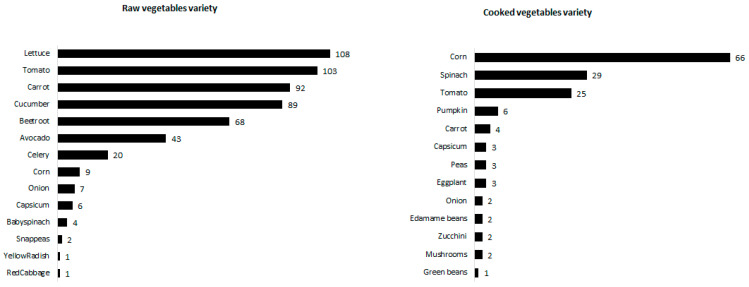
Variety of vegetables (numbers represent the number of menus each vegetable was mentioned).

More items contained raw (16.9 ± 10.3 items) than cooked vegetables (8.7 ± 6.8 items, *p* < 0.01) and variety was larger for raw (4.9 ± 1.8) than for cooked (1.3 ± 1.2; *p* < 0.01) vegetables. Across all menus, 14 raw and 13 cooked vegetables were mentioned (Figure 3). Raw vegetables were mostly lettuce, cucumber, tomato and beetroot, and used in salads and sandwiches. Celery, carrot and cucumber were also often listed as snacks (with or without dips). Corn on the cob was the most listed cooked vegetable, followed by spinach and tomato. Other cooked vegetables were very infrequently mentioned.

The only statistically significant difference between schools of different SES levels was that high SES schools offered more vegetable-containing hot meals/foods than low SES schools (51.0% versus 42.1%; *p* = 0.019). Additional ANCOVA analysis showed that the proportion of vegetable-containing hot meals/foods was still influenced significantly by SES (*p* = 0.039). Looking at school size, larger schools had significantly larger proportions of total vegetable-containing items offered, vegetable-containing hot meals/foods and vegetable-containing snacks than smaller schools (Table 2). School size had a large impact on the number of offerings; small schools had fewer menu items than medium and large schools and fewer vegetable-containing items, which was also visible in some subcategories (data not shown). As the proportion of government (*n* = 90) and non-government (*n* = 22) schools was unbalanced and school size was associated heavily with school type (15 of the 22 non-government schools were small schools), only significant effects based on ANCOVA analysis are indicated in Table 2. The analysis showed that government schools offered a significantly larger proportion of vegetable-containing snacks (*p* = 0.021) and vegetable-containing meal deals than non-government schools (*p* = 0.032).

## 4. Discussion

This study quantified the number and proportion of vegetable-containing items on online primary school canteen menus. It showed that over two-thirds of menu items did not contain any amount of vegetables. Specifically, most sandwiches and hot foods did not include vegetables, and there were no beverages containing vegetables. Furthermore, the variety in vegetables offered was quite small, particularly for cooked vegetables, and 67% of Crunch&Sip (C&S) packages did not provide any vegetable options. Some differences between schools were found based on school size, but few based on SES and school type.

To our knowledge, this is the first audit specific to vegetables in primary school canteens globally. Other canteen menu studies focused on limiting unhealthy practices in school canteens by studying the adherence to policy guidelines [17,18,19,20,21,22] or focused on specific nutrients [31] or frequency of food groups [32]. This last study allocated menu items to food groups to measure compliance to French guidelines for healthy school menus. Unfortunately, this study did not report vegetable-specific data, so no comparison between countries could be made. Myers et al. focused mostly on adherence to WA’s school food-service policy and reported whether menus offered different types of raw vegetables [18]. Both our study and Myers’ research found similar results for sandwiches with vegetables (offered by 96% and 99% of schools) and salads (83% and 79%), but more schools in our study offered raw vegetable snacks (53% versus 42%). The results point to differences between states within Australia in vegetable offerings through canteens. This current study provides novel data on the availability of vegetables relative to other foods on Australian primary school canteen menus, which can be used as the first step to design strategies to increase children’s exposure to and demand for vegetables in the school environment. This can help to overcome the gap between children’s vegetable consumption and dietary guidelines.

Smaller schools offered a lower proportion of vegetable-containing items, which could be explained because they may have fewer resources available, and preparing vegetables is considered labour-intensive. Very few differences were found between SES levels and school types. Thus, despite overall better diet quality among high SES groups [25], this does not coincide with increased vegetable offerings in school canteens. Similarly, despite non-government schools not needing to adhere to school food-service guidelines, this does not translate into fewer vegetable offerings. These findings indicate that tailored strategies are not necessary at this stage, as overcoming barriers of resource and time constraints would benefit schools of all sizes.

The findings implicate there are plenty of opportunities to increase the vegetable offering in primary school canteens. It is difficult to objectively quantify a desirable percentage of vegetable-containing items on menus as there will always be items where adding vegetables is not needed or reasonable, e.g., plain milk. However, for certain menu categories, it would seem desirable that all foods contain vegetables, e.g., wraps, sandwiches and hot meals. This would improve vegetable offering considerably, as most sandwiches and hot foods did not include vegetables. There were no beverages or frozen items with vegetables on the menus, whereas they are commercially available in retail already.

Meal deals provide another opportunity to increase vegetable supply. Only a few meal deals contained a vegetable side dish. A meal deal containing a vegetable side dish could be an attractive item for parents to order, as vegetables are most commonly consumed as part of a meal [33]. Another strategy is to include vegetables in C&S offerings, as in the current study, 67% of C&S items did not include vegetables but only offered fruit. This would corroborate with the intentions of the C&S program in WA, which specifically promotes the consumption of vegetables over fruit [29]. Due to their low energy density, vegetables do not comprise a complete meal on their own. In order to meet children’s energy needs and parental budgets, vegetables could be presented as part of an integrated meal, e.g., as a salad that contains a protein component.

When offering vegetables, it is important to realise that children’s food consumption is driven by acceptance [34], and therefore vegetable-containing foods and dishes offered in canteens should be well accepted by children. As vegetables do not possess sensory characteristics aligned with our innate likes [35], most vegetables will be better accepted when consumed as part of meals or accompanied by food items from different food groups rather than consumed on their own. Consumer sensory testing with children could be undertaken to guarantee acceptance of menu items and may limit the number of vegetables to include in a dish.

Ideally, increasing vegetable offerings would not affect the workload of canteen operators. Potential solutions could be using pre-cut vegetables for hot meals, sandwiches and snacks, or reducing the number of items on menus which would improve preparation efficiency, and scale efficiency might reduce costs. It could be argued that this strategy would consequently lead to fewer vegetable-containing items on the menus, but if the focus of canteen policies were to promote vegetable intake, this risk would be negated.

Some limitations of this study need to be noted. The use of taste.com.au to decide whether an item contained vegetables when recipe information was not available can be disputed. This method was selected as contacting schools was beyond the study’s scope and would be impractical to conduct at a large scale for ongoing monitoring. Due to this limitation, this study did not consider the actual vegetable content within menu items nor considered sales. Hence, no conclusions were drawn on the actual amount of vegetables being supplied or sold by school canteens. Lastly, this study was limited to schools in the Greater Sydney area, which limits external validity. Most Australians live in metropolitan areas, but schools in rural areas might have different access to fresh vegetables. Because of these limitations, this study should be seen as a preliminary study.

Our study provides baseline audit data that can be used as a comparison for future research. The current systematic method can be applied to audit vegetable offerings in different Australian states or assess differences between urban and rural areas. All Australian states and territories currently have canteen policies mandating the provision of healthy foods and, as such, promoting vegetable offerings indirectly. However, there are no *specific* criteria for vegetables, e.g., a certain proportion of dishes/items needing to contain vegetables. Larger scale audits can help inform policies to set such standards, and their effect can be assessed using the current methodology. The current methodology can also be applied to countries with similar school food systems (e.g., New Zealand). It is recommended for future research to quantify the actual amount of vegetables being offered to, and purchased by, students in primary school canteens.

## 5. Conclusions

In conclusion, primary school canteen menus offer a large variety, but only a third of items contained vegetables. Sandwiches, hot foods, snacks and Crunch & Sip packages are categories where vegetables seem underrepresented. School size influenced vegetable offering, whereas there was very little influence of SES and school type on vegetable offering, indicating that tailored strategies are not necessary at this stage, as overcoming barriers of resource and time constraints would benefit schools of all sizes. By knowing the current state of play, new opportunities can be developed to increase the vegetable supply in schools and improve children’s health in the long term.

## Figures and Tables

**Table 1:** Mean (and standard deviation, SD) of total number of menu items, number of items with vegetables and proportions of items with vegetables (%) within each menu category.

		Total Number of Items (*n*)		Number of Items with Vegetables (*n*)		
Category	Number of Menus Listing the Category (*n*) ^1^	Mean	SD	Mean	SD	Proportion of Items with Vegetables (%)
Overall	112	80.4	34.0	25.7	15.8	30.4%
Beverages	112	10.8	4.5	0	0	0.0%
Hot meals/foods	112	22.1	9.7	10.6	5.8	46.2%
Sandwiches	110	16.1	7.9	7.2	5.6	39.7%
Salads	93	5.8	3.8	5.8	3.8	100.0%
Snacks	111	23.7	12.3	2.4	3.9	7.9%
Meal deals	41	6.5	4.9	3.2	2.5	50.5%

^1^ Based on the number of menus that listed the subcategory.

**Table 2:** Mean (and standard deviation, SD) proportions of items with vegetables (%) within each menu category split for school size (≤400 students = small; 401–600 students = medium; >600 students = large) and school type (government and non-government).

	Small		Medium		Large		Government		Non-Government	
	Mean	SD	Mean	SD	Mean	SD	Mean	SD	Mean	SD
Overall	27.8 ^b^	8.5	32.0 ^ab^	8.5	33.3 ^a^	7.8	30	8.8	31.9	7.8
Hot meals/foods	43.1 ^b^	14.4	44.6 ^ab^	15.1	52.0 ^a^	11.3	46.3	14.4	45.9	13.5
Sandwiches	35.3	19.3	45.7	14.5	42.6	17.2	39.4	18.4	41.1	17.4
Salads	100	0	100	0	100	0	100	0	100	0
Snacks	6.2 ^b^	8.5	7.0 ^ab^	9.4	10.9 ^a^	8	8.8	9.1	3.7 *	5.2
Meal deals	55.9	27.4	43.1	34.2	49.7	19.4	53.9	26.4	30.5 *	23.9

No superscript letters or symbol indicate one-way ANOVA did not find any main effects and therefore no post-hoc pairwise comparison was conducted for these variables. ^ab^ Letters indicate significant differences between groups (post-hoc pairwise comparison following one-way ANOVA, Bonferroni corrected *p* < 0.05). Same letters mean no significant differences between means. * Indicate the significantly lower means of values found in non-government schools compared with government schools (ANCOVA, *p* < 0.05).

## Data Availability

The data presented in this study are available on request from the corresponding author. The data are not publicly available due to being collected as part of a contracted project.
